# School-Based Eating Interventions—Are Students Eating Healthily?

**DOI:** 10.3390/nu16183081

**Published:** 2024-09-13

**Authors:** Mariana Calle, Elinor Fondell

**Affiliations:** Health Sciences Department, Worcester State University, Worcester, MA 01602, USA; efondell@worcester.edu

School nutrition is an important key modifier in terms of child and adolescent nutrient intake. A poor diet early in life can lead to a multitude of immediate and long-term health problems. Changes to the dietary information given in the school setting as well as changes to the food programs offered have the potential to promote adherence to a healthy diet, which can lead to lifelong health benefits.

The present Special Issue includes two multicomponent school-based nutrition interventions to increase fruit (F) and vegetable (V) intake in children [1,2], an evaluation of the impact of the new Child and Adult Care Food Program meal and snack menu patterns in children attending childcare centers [3], a qualitative study assessing participation facilitators and barriers for emergency school meals and the pandemic electronic benefits (P-EBT) [4], and one cross-sectional study evaluating factors related to the use of caffeine-containing energy drinks in middle school students [5].

Both multicomponent school-based interventions in the present Special Issue used the well-known theory of planned behavior as one of their theoretical models. More specifically, attitude, subjective norms, and perceived behavioral control can predict behavioral intentions. This approach has effectively predicted and changed diet-related behaviors and intentions in youth [6]. Increased awareness of the parental role in children’s eating behaviors is warranted. For example, parents may not know that children need to be repeatedly exposed before they accept unfamiliar flavors or/textures. Hence, they stop offering that food [7].

The Dutch “Kokkerelli learning street” program combined the classroom with experiential learning strategies. Using this program, Hahnraths et al. also examined FV preferences, knowledge, attitudes, and intention to consume FV short term (directly after the intervention) and after 3 months [1]. The 3-week interventions showed positive results for all determinants, except intention in the short term, but no improvements after 3 months. In this study, the authors combined FV. The intervention intensity and the lack of parental involvement (e.g., there was no homework) may explain the lack of sustainability of the positive short-term effects of participating in this program. Hahnraths et al. suggested separating F from V and including the parents to participate in the intervention. Interestingly, this was addressed in the other article from this Issue. In “Nutri-skolica”, the school-based 3-year intervention by Ilić et al., parents received online education and participated with their children through the assigned homework throughout the intervention [2].

Both of the school-based nutrition interventions included in the Special Issue aimed to change F and V intake in children aged approximately 7–10 years old. Preference is the main factor associated with F and V in children, whereas Vs are not an innate preferred food. However, participation in the 3-year intervention had a stronger effect on changing FV intake than change in FV preference among primary school children [2].

Overall, these intervention studies [1,2] emphasize critical points for successful future interventions, such as the length and intensity of the intervention, and the experiential nature of programs, including cooking classes and /or taste tests. Furthermore, the frequency of exposure to F and V is important, in addition to parents’ involvement as positive reinforcers of eating FV in the context of the school-based interventions.

It is noteworthy that the 3-year intervention “Nutri-skolica” was not fully implemented due to the COVID-19 pandemic. On the same note, the next study in the Special Issue examines the changes due to COVID-19 regarding the implementation of emergency school meals and pandemic electronic benefits in an urban setting. Cadenhead et al. presented qualitative data on facilitators and barriers to using the available emergency school meals and the P-EBT [4]. The researchers identified key facilitating sub-themes such as clear communication, easily accessing sites, and high variety. More students may have participated in the grab-and-go if there had been greater awareness of the program and fewer barriers to accessing the meals. Parents used the P-EBT, but some expressed difficulty navigating the system. Understanding low program participation is key to improving the accessibility of available assistance, especially considering its impact on increasing food security in vulnerable populations. Even after the pandemic, addressing barriers to low summer meal participation will aid in reducing food insecurity.

Through the Hunger-Free Kids Act of 2010, the US Department of Agriculture (USDA) established policies to improve the nutritional quality of food and beverages served to US children through federal food assistance programs and it made changes in the Child and Adult Care Food Program (CAFP). In this Special Issue, Dave et al. assess what changes in children’s dietary behaviors occurred as a result of the new CACFP meal pattern requirements [3]. The findings suggest that providers may need more assistance meeting the standards on calories from fat and saturated fat. The authors concluded that children’s dietary intake improved on only some of the domains targeted by the revised CACFP meal patterns—specifically whole grains, milk, and juice [3].

The Smart Snacks rule was part of the implementation of the 2010 Act, which allows the USDA to regulate foods and beverages sold in schools outside of the school meal programs. For example, energy drinks are not permitted in schools, and a study using data from the School Nutrition and Meal Cost Study reported 84% compliance in middle schools in the United States [8]. In this Special Issue, a cross-sectional study conducted in Japan showed that boys were more likely to consume energy drinks than girls [5]. Buying their own snacks, not understanding nutritional labels on foods, consuming highly caffeinated beverages, late bedtimes on weekdays, waking up at about the same time every day, and higher body weight were factors associated with the use of energy drinks in Japanese boys.

The National School Lunch Program (NSLP) and the School Breakfast Program have to follow specific nutrition requirements consistent with the Dietary Guidelines for Americans. Eating school breakfast and school lunch every day was associated with modestly healthier dietary intakes in US schoolchildren [9]. This is consistent with a recent review comparing the nutritional value of lunches brought from home to those provided by the NSLP [10]. The authors provided quantitative and qualitative evidence that a significant proportion of school-aged children who brought their lunch from home consumed less nutritionally balanced meals [10].

Taken together, the studies presented in this Special Issue highlight the relevance of the role of schools in children’s nutrition. The school environment represents a unique opportunity to positively impact the nutrition of children considering they can consume a significant percentage of their daily intake there. Similarly, a recent systematic review concluded that FV interventions provide a promising avenue by which children’s consumption can be improved. Future interventions should place more focus on vegetable intake [11].
